# Legalized

**Published:** 1891-10

**Authors:** 


### Legalized.

The Metric System has been legalized in the following countries: Argentine Confederation, Austria, Belgium, Bolivia, Brazil, Chili Columbia, Ecuador, France, German Empire, Greece, India, Italy, Mexico, Netherlands, Peru, Russia, Spain, Uruguay.

Rules for Bathing.—It is well to commence with these baths as soon as the first infirmities of age begin to make themselves felt between the 50th and 60th year.

Two to three baths should be taken every week. As the water cools off, hot water must be added and the thermometer consulted.

The best time for bathing is in the forenoon, about two hours after breakfast, or the afternoon, about four hours after the midday meal.

After the bath the body must be well dried and rubbed with coarse towels.

Baths either too hot or too cold are dangerous to old people.

